# Unique Properties of Eukaryote-Type Actin and Profilin Horizontally Transferred to Cyanobacteria

**DOI:** 10.1371/journal.pone.0029926

**Published:** 2012-01-10

**Authors:** Arthur Guljamow, Friedmar Delissen, Otto Baumann, Andreas F. Thünemann, Elke Dittmann

**Affiliations:** 1 University Potsdam, Institute for Biochemistry and Biology, Department of Microbiology, Golm, Germany; 2 Bundesanstalt für Materialforschung und -prüfung Federal Institute for Materials Research and Testing, Berlin, Germany; 3 University Potsdam, Institute for Biochemistry and Biology, Department of Animal Physiology, Golm, Germany; University of Queensland, Australia

## Abstract

A eukaryote-type actin and its binding protein profilin encoded on a genomic island in the cyanobacterium *Microcystis aeruginosa* PCC 7806 co-localize to form a hollow, spherical enclosure occupying a considerable intracellular space as shown by *in vivo* fluorescence microscopy. Biochemical and biophysical characterization reveals key differences between these proteins and their eukaryotic homologs. Small-angle X-ray scattering shows that the actin assembles into elongated, filamentous polymers which can be visualized microscopically with fluorescent phalloidin. Whereas rabbit actin forms thin cylindrical filaments about 100 µm in length, cyanobacterial actin polymers resemble a ribbon, arrest polymerization at 5-10 µm and tend to form irregular multi-strand assemblies. While eukaryotic profilin is a specific actin monomer binding protein, cyanobacterial profilin shows the unprecedented property of decorating actin filaments. Electron micrographs show that cyanobacterial profilin stimulates actin filament bundling and stabilizes their lateral alignment into heteropolymeric sheets from which the observed hollow enclosure may be formed. We hypothesize that adaptation to the confined space of a bacterial cell devoid of binding proteins usually regulating actin polymerization in eukaryotes has driven the co-evolution of cyanobacterial actin and profilin, giving rise to an intracellular entity.

## Introduction

The actin family of proteins is an evolutionary ancient group whose signature feature, the polymerization into filaments, is the basis for a remarkable functional versatility and the resultant extensive prevalence of actins in the living world [1], [2]. Long believed to be restricted to eukaryotes and despite their very low sequence identity of ∼14% with each other and eukaryotic actin, prokaryotic actin homologs have been identified through structure-based alignments [3]. ActM, an actin homolog found solely in a strain of the cyanobacterium *Microcystis aeruginosa* stands out as it shows a considerable sequence identity (65%) with eukaryotic actin. It is encoded in direct proximity to PfnM, which is, with an identity of 84%, the only known homolog of the eukaryotic actin binding protein profilin in prokaryotes. ActM and PfnM are a very clear example of otherwise rarely documented cases of eukaryote-to-prokaryote horizontal gene transfer [4], [5], [6]. ActM appears to have adopted a structural function as it is part of a shell-like layer localized towards the periphery of the cell [7].

In eukaryotes, cytoplasmic actin is an essential protein that is the building block of the microfilament cytoskeleton establishing an extended internal scaffold essential for many fundamental cellular functions [8], [9], [10]. To control actin network architecture, eukaryotes employ more than 100 actin binding proteins (ABPs) generally falling in two classes with either actin monomer or filament binding properties [11]. One member of the first is profilin, which binds actin in a strict 1∶1 molar ratio and facilitates its polymerization by shuttling monomers to elongating filament ends where actin binds and profilin is released from the growing polymer [12], [13].

The numerous interactions of ABPs with actin are believed to be responsible for the evolutionary constraint on its sequence, making it one of the most conserved proteins [1]. To date, homologs of the eukaryotic ABPs have not been identified in bacteria. This may have contributed to the high degree of prokaryotic actin sequence diversion. For instance, the ParM and AlfA proteins involved in plasmid segregation are believed to have only one protein binding partner [14], [15]. The actin homologs MamK, FtsA and MreB are each involved in key physiological processes: while MamK is responsible for the alignment of intracellular magnetic vesicles in magnetotactic bacteria [16], [17], FtsA plays a role in the proper localization of components of the cell division machinery [18] and MreB is essential for cell stability and shape determination [19], [20], [21]. Although some binding proteins are known for these actins [17], [22], [23], the degree of complexity of the eukaryotic actin-ABP network is unrivalled by prokaryotes.

The cyanobacterial ActM and PfnM provide the rare opportunity to assess the functional plasticity and adaptive flexibility of a naturally occurring actin/profilin pair detached from the influence of native eukaryotic ABPs thus shedding light on the co-evolution of one of the most highly conserved proteins and its closely associated binding partner. Therefore, the aim of the present study was the biochemical and structural characterization of both proteins to determine which of their well-known eukaryotic characteristics may be conserved, modified or lost, thus possibly displaying new, unusual properties. Centered on the key feature of actin polymerization the goal of our experiments was to determine detailed structural parameters of potential ActM aggregates, the influence of PfnM on the assembly process and the possible functional implications in a bacterial host organism.

## Results

### ActM and PfnM interact and form a shell-like, hollow enclosure in vivo

**Figure 1:**
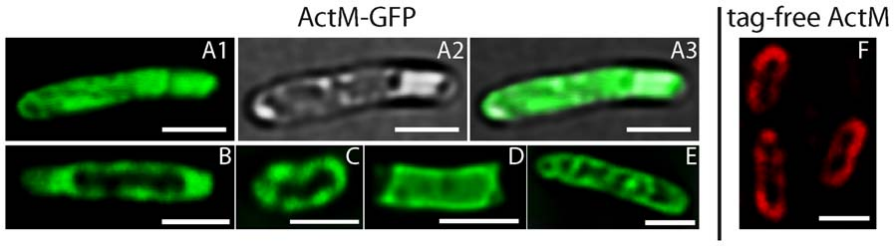
ActM-GFP expression in *E.coli.* ActM-GFP adopts a variety of shapes and apparently is not freely diffusible in *E.coli*. All images show the GFP-channel, except for A2 and A3 which display the transmission channel and an overlay of transmission and GFP, respectively. Image F shows anti-actin/TRITC immunofluorescence of untagged ActM expressed in *E.coli*. Scale bars: 2 µm.

Anti-actin immunofluorescence microscopy has revealed a distinct ActM localization towards the cell's periphery visible as rings in optical sections, equivalent to a shell-like intracellular structure, indicating a structural function of ActM (ref. [7] and Figure S1). In the absence of a reliable system for genetic manipulation and stable heterologous expression of proteins in *Microcystis*, we carried out further investigations with GFP fusions of ActM and PfnM in *E.coli.* In more than 90% of the cells observed, ActM-GFP is unevenly distributed with the protein being occluded from various intracellular locations (Figure 1). A common pattern is an accumulation to the peripheral parts of the cell (Figure 1 B, C, E), reminiscent of the ring-like distribution of ActM in *Microcystis aeruginosa* (Figure S1). This was the only pattern we also found in immunofluorescence micrographs of *E.coli* cells heterologously expressing untagged ActM (Figure 1F). However, ActM-GFP also condenses into elongated shapes of varying width running parallel to the cell's longitudinal axis or traversing the cylindrical cytoplasm (Figure 1 A1-3, E). The different ActM-GFP patterns do not appear in fixed ratios, in fact the ratios seem to vary with culturing conditions and incubation time. Presumably, the observed shapes undergo constant interconversion. The heterogeneous intracellular occurrence of ActM-GFP suggests the formation of higher-ordered accumulations of the protein whose size prevents their free diffusion through the cytoplasm. However, the emergence of these ActM-GFP accumulations is not influenced by the presence of PfnM since we found the distribution patterns of ActM-GFP to remain unaltered in cells co-expressing PfnM.

**Figure 2:**
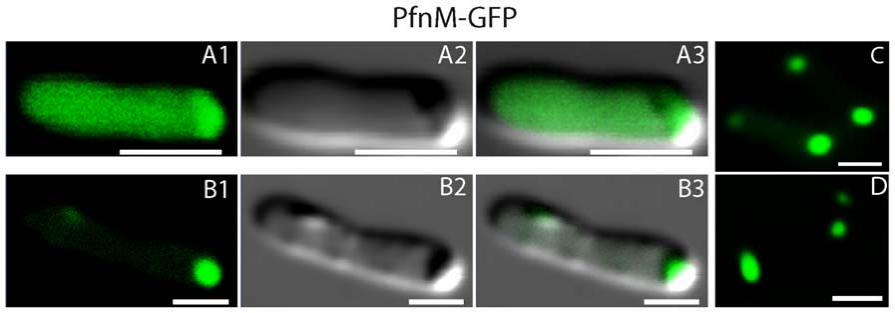
PfnM-GFP expression in *E.coli.* In *E.coli*, PfnM-GFP distributes evenly in the cytoplasm (A1-3) or is localized to the cell poles (B1-D). Scale bars: 2 µm.

**Figure 3:**
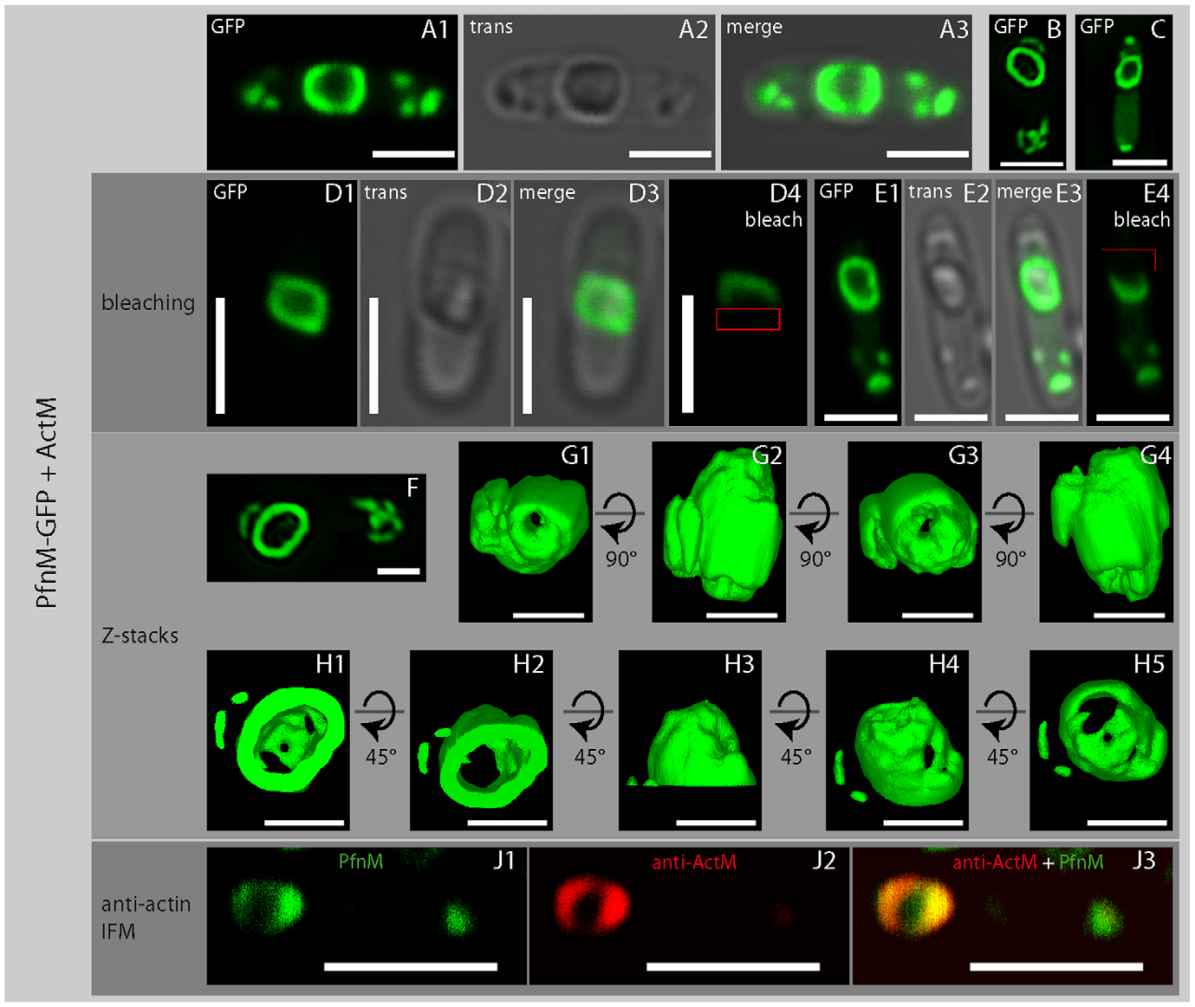
A hollow enclosure in cells co-expressing PfnM-GFP and ActM. Co-expression of PfnM-GFP and ActM gives rise to a hollow compartment. This enclosure is not dynamically rearranged, as fluorescence does not recover 30 minutes after bleaching (D4, E4, the bleached region is indicate by a red rectangle). Z-sectioning and 3D reconstructions of the cell shown in F. Stepwise rotations along the x-axis of the total enclosure (G1-4) or its “top” half (H1-5) is shown. Immunodetection of ActM in the enclosures of *E.coli* expressing both ActM and PfnM-GFP reveals a co-localization (J1-3). Images show either GFP-channel (“GFP”), transmission image (“trans”) or an overlay of both (“merge”). Scale bars in Z-sectioning and 3D reconstruction: 1 µm. All other scale bars: 2 µm.

The intracellular distribution of PfnM-GFP shows two distinct patterns, a homogenous cytosolic dispersion and a concentration in polar foci, frequently also a combination of both (Figure 2). The co-expression of PfnM-GFP and ActM gives rise to large intracellular structures that appear ring-like in two-dimensional micrographs (Figure 3). Z-sectioning and subsequent 3D reconstruction show that these rings represent hollow, shell-like enclosures that span the cell's whole width making extensive contact with its inner boundaries (Figure 3 F-H). After bleaching distinct regions of the enclosures with high laser intensities, fluorescence is not recovered for at least 30 minutes indicating that the enclosures are not dynamic and PfnM-GFP is stably attached (Figure 3 D4 and E4). Since we have never observed these structures in any other cases of heterologously expressed GFP fusions, we believe them to be the consequence of a specific interaction between PfnM and ActM. To exclude the possibility that we observed an artifact resulting from unspecific aggregation and deposition in inclusion bodies [24], we estimated the amount of protein found in the soluble and the insoluble fraction of these cells. The soluble form represents nearly all of the cellular ActM content and well more than half of all PfnM-GFP (Figure S2). We frequently observed small, compact GFP foci (Figure 3 A, B, C, E) possibly representing inclusion bodies being responsible for the detected insoluble protein.

The observed enclosure probably requires at least one structural component as a foundation of a stable PfnM-containing proteinaceous body. ActM, although soluble in the cytoplasm, may be absent from the actual structure and mainly be required for a putative assembly process of PfnM. More likely, however, given the known ability of actins to polymerize, ActM itself aggregates and in combination with bound PfnM provides the framework to form a structural intracellular entity. To determine whether ActM is present in the observed structures we used a polyclonal anti-actin antibody to perform anti-actin immunofluorescence microscopy with cells co-expressing PfnM-GFP and ActM. In cells carrying the enclosure we found ActM in the characteristic ring-shaped pattern along the outline of the structure (Figure 3 J1-3). The respective fluorescence signals originating from PfnM-GFP and anti-actin immunostaining overlap considerably indicating a co-localization of PfnM-GFP and ActM in the intracellular enclosure. Thus, we speculate that PfnM binding to ActM may play an important role during the assembly of the intracellular structure.

### ActM and PfnM bind to each other

**Figure 4:**
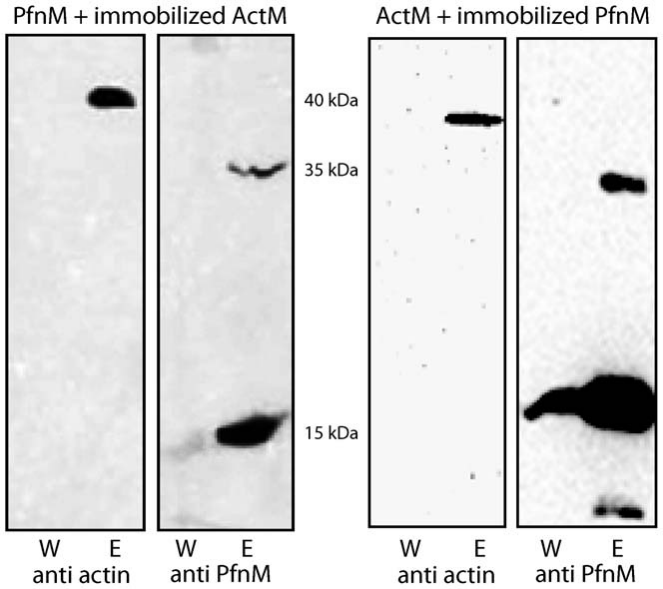
Binding and co-elution of ActM and PfnM. One potential binding partner was immobilized, the other added in solution. Protein blots and immunodetection of final wash (W) and eluate (E) are shown; employed antibody is indicated at the bottom. Molecular weights are 39 kDa for ActM and 17 kDa for PfnM.

Given the conservation of binding sites in both proteins [7], we wanted to determine experimentally whether monomeric ActM can bind to PfnM. In a co-elution assay, one of the proteins was immobilized through a fused His-tag to a Ni-NTA agarose matrix while the other was untagged and added in solution. After thorough washing, immobilized proteins were eluted and the eluates analyzed on protein blots. We found both ActM and PfnM in the eluates of reciprocal setups, with the mobile protein absent from the final wash (Figure 4), showing that monomeric ActM and PfnM specifically bind each other. In equivalent experiments PfnM co-eluted with rabbit G-actin, suggesting the conservation of the actin-profilin interface (Figure S3).

We also found that PfnM-GFP co-elutes with ActM, (Figure S4 A) and that ActM-GFP does not bind PfnM (Figure S4 B), explaining the absence of a PfnM effect on ActM-GFP distribution in *E.coli*. Apparently, the fusion of the GFP protein to ActM impairs interactions with potential binding partners. Using N-terminal GFP-ActM fusion proteins did not abolish these problems as we also neither observed a PfnM effect on intracellular GFP-ActM localization nor did the employed antibody detect GFP-ActM on immunoblots.

### ActM forms polymeric, ribbon-shaped filaments

**Figure 5:**
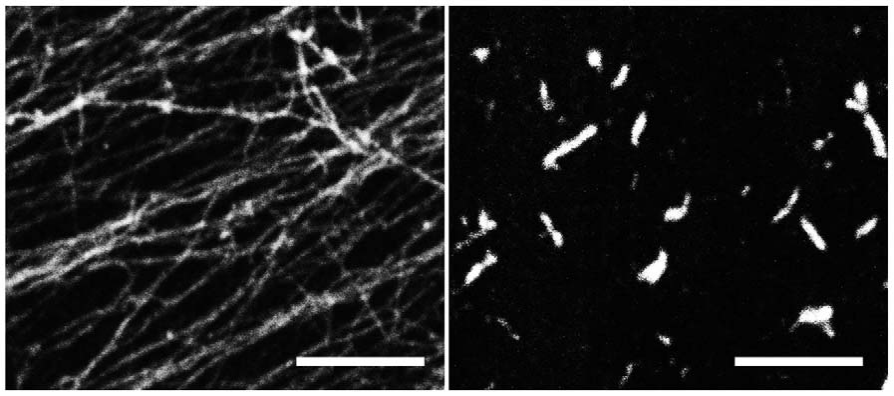
Phalloidin staining of polymerized rabbit actin and ActM. Rabbit actin (left) polymerizes into long filaments forming an interwoven network. ActM polymers (right) appear as short filaments assembling in bundles and sheets. Scale bars: 5 µm.

A prerequisite for ActM to form an intracellular compartment-like body would be its property to self-aggregate. We have used fluorescent derivatives of the F-actin binding drug phalloidin [25] with actin from rabbit skeletal muscle and with purified ActM. Rabbit actin forms a dense network of thin filaments (Figure 5, left) which are usually more than 10 µm long and are likely to be in the 100 µm range. Under the same conditions, ActM forms short filaments of 0.5–2.0 µm that cluster together to give rise to extended, sheet-like structures (Figure 5, right). The specificity of the phalloidin/F-actin binding provides a strong argument that ActM has retained the ability to polymerize.

For detailed structural insight, we measured the small-angle X-ray scattering (SAXS) of solutions of polymerized ActM in the size range of 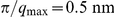 to 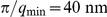. While the determination of the much larger total length of F-actin filaments is not possible with this method, valuable information on the filament cross sections is available from these data (note that SAXS produces intrinsically representative statistical sample averages). For data interpretation, we applied both curve fits of model functions [26] and model-independent methods [27].

**Figure 6:**
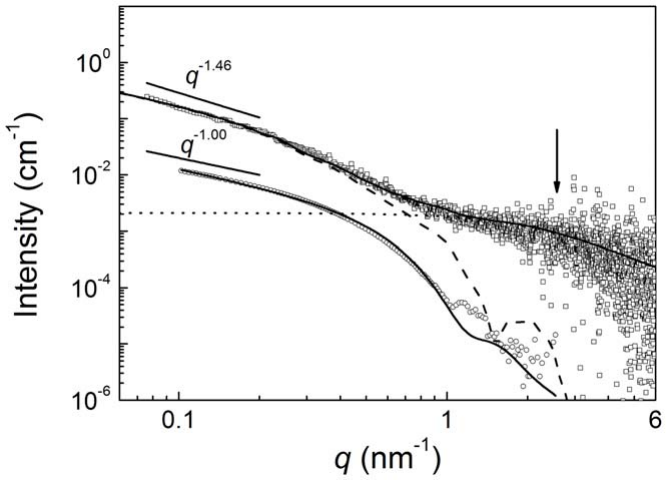
SAXS data of polymerized ActM and rabbit actin. Data of rabbit F-actin (circles) are from [29]. Best fit curves (solid lines) are from a cylinder function for rabbit actin with a radius of *R* = 3.0 nm and a parallelepiped for ActM (squares) with *c* = 4.2 nm and *b* = 15 nm. Length of cylinder and parallelepiped where held constant for fitting at 500 nm. The power-law scalings at low *q* are indicated as straight lines. The arrow points at the *q*-region where the scattering of short random-coil structures is visible. Contributions of the parallelepiped and the random coil structure are displayed (dashed and dotted line, respectively).

Small-angle scattering patterns of eukaryotic F-actins [28] are typically described by model scattering curves of long cylindrical rods. As a signature of this geometry, their intensity decays proportional to *q* ^−1^ at low *q*-values while the steep bending in the higher *q*-range is characteristic for the rod's radius. As a reference, we analyzed available small-angle neutron scattering data of rabbit F-actin ([29] Figure 6, circles). We fit the data sufficiently by using the cylindrical model [26] with a filament radius of 3.0 nm (Figure 6, circles and solid line). A polydispersity of 0.2 was applied to take into account that the thickness of F-actin is not constant along the filament [30]. The *q* ^-1.00^ scaling at low *q*-values is characteristic for a cylindrical shape with an estimated cylinder radius of 2.6 nm [29]. A solid cylinder has recently also proven very useful as an initial reference volume for the construction of an F-actin high resolution structure based on electron cryomicroscopy data [9]. In summary, the shape of the rabbit F-actin scattering curve could be interpreted sufficiently with the simple cylinder as a low resolution structure model.

While the SAXS pattern of ActM confirms the polymerization observed microscopically (Figure 6, squares), it is not possible to fit the cylinder model sufficiently to the ActM data. Instead of the expected *q* ^-1.00^ scaling of a cylinder, the intensity scales with *q* ^-1.46^ in the low *q*-range, indicating a significantly pronounced ribbon-shaped filament structure for ActM. Note that an extended sheet scales with *q* ^−2.00^ [29]. In addition to that, the intensity around 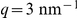 is higher than must be expected for a compact filament structure (Figure 6, arrow). According to Porod's law [31] a compact structure should display a region where the intensity decays proportional to *q* ^−4^, i.e. the curve must decay much steeper than observed here, leading to the assumption that small structure entities with sizes of around 1 nm are also present in the polymer. Tentatively, we assume short random-coil structures for interpreting the scattering contribution around 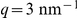 and thus the total scattering could be described as a sum of filament and random-coil scattering as



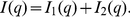
(1)


Ribbon shaped filaments can be modeled as parallelepipeds [32] of length *a*, width *b* and thickness *c* with *a* > *b* > *c*. Their scattering function is given by [33]



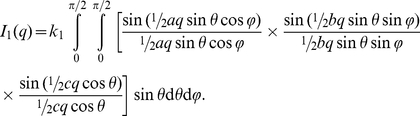
(2)


The scattering of a polymer with a random coil structure is described by the Debye formula



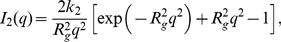
(3)


where *R*_g_ is the ensemble average radius of gyration. The *k*_1_ and *k*_2_ are scaling factors. The best fit of eq. (1) to the data of polymerized ActM (Figure 6 squares and solid line) results in a height of 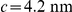 and a width of 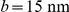
for the cross section dimensions of the filament. As the filaments' length is beyond the maximum size accessible it was held constant at 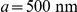. The radius of gyration for the random-coil contribution to the scattering is 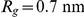 from which we determined the corresponding number of amino acids as follows: For proteins with random-coil behavior, *R*_g_ obeys the power-law relationship of linear flexible polymer chains



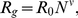
(4)


where *N* is the number of amino acids of the chain, *R*_0_ is a constant and ν is an exponential scaling factor. Kohn et al. reported values of 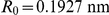 and 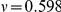 in a study on the random coil behavior of unfolded proteins [34]. Using these values, we calculated from eq. (4) that the random coil segments of polymerized ActM consist of 7.6 to 9.7 amino acids.

### PfnM binds to polymeric ActM and induces filament bundling

**Figure 7:**
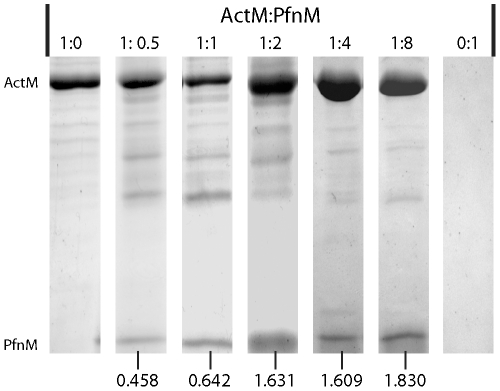
Polymerization and ultracentrifugation of ActM and PfnM. SDS-PAGE of pellet fractions of 7 reactions containing ActM and PfnM in different ratios (indicated at the top) after ultracentrifugation at 100,000 × g. PfnM (17kDa) co-sediments with polymerized ActM (40 kDa), the relative amount of PfnM in the pellet is indicated at the bottom.

Binding of profilin can influence the polymerization properties of actin [35]. Therefore, we initiated polymerization in solutions containing ActM and PfnM in the ratios of 1∶0, 2∶1, 1∶1, 1∶2, 1∶4, 1∶8 and 0∶1 and subsequently ultracentrifuged these at 100,000 × g. We analyzed the resulting pellet fractions with respect to their ActM and PfnM content by SDS-PAGE (Figure 7). A 17 kDa band was present in all reactions that contained both ActM and PfnM. No such band was observed in set-ups devoid of either ActM or PfnM. This shows that ultracentrifugation of polymerized ActM alone does not result in the appearance of a 17 kDa band. Additionally, this proves that purified PfnM cannot be pelleted by ultracentrifugation at 100,000 × g. Rather, the results indicate that PfnM binds to polymerized ActM and can be co-pelleted by ultracentrifugation. For a rough estimate of the stoichiometry of ActM and PfnM in the pellet fraction, we measured the staining intensities of individual bands after Coomassie-staining (Figure 7). The molar ratios of ActM:PfnM in the pellets were 1∶0.458 (for the reaction initially containing ActM and PfnM in a 2∶1 ratio), 1∶0.642 (1∶1), 1∶1.631 (1∶2), 1∶1.609 (1∶4) and 1∶1.830 (1∶8). This shows that the binding of PfnM to ActM filaments is concentration-dependent and reaches saturation at approximately 2-fold molar excess of PfnM. In control experiments with rabbit actin, PfnM was detectable only in set-ups that contained at least a 4-fold molar excess of PfnM. At an initial actin:PfnM ratio of 1∶4, the pellet ratio was 1∶0.475 (Figure S5 A).

**Figure 8:**
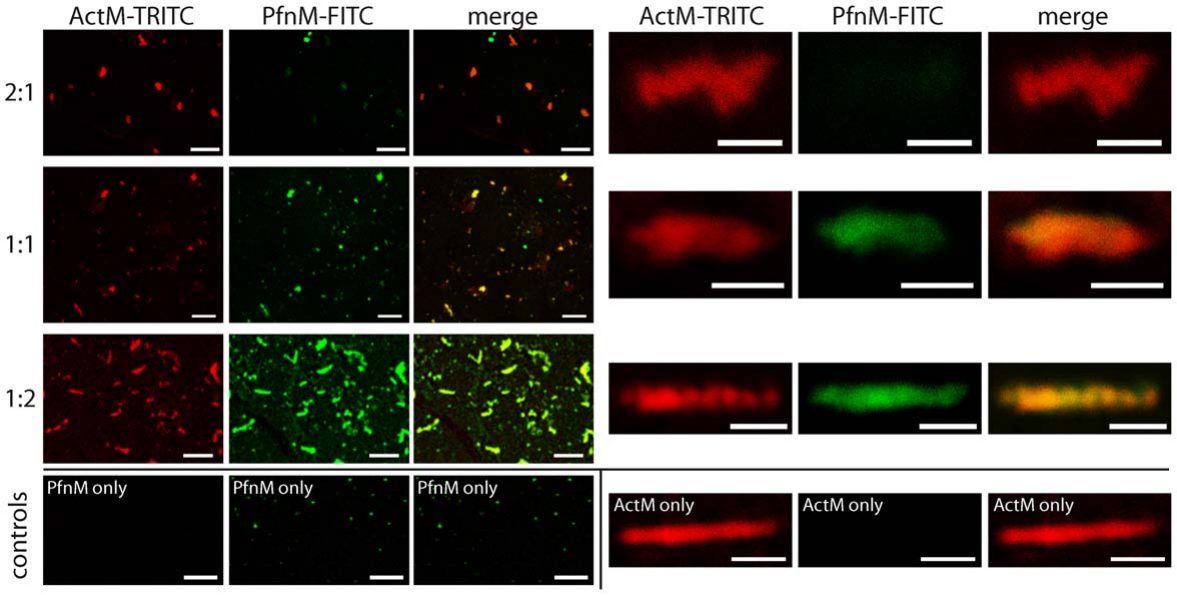
Fluorescence microscopy of PfnM binding to ActM polymers. FITC stained PfnM (green) co-localizes with phalloidin-TRITC stained ActM polymers (red). Single ActM bundles are covered with PfnM along their length. Fluorescence signals increase with increasing PfnM concentration, molar ratios of ActM:PfnM are indicated. Images on the left hand side show an overview (scale bars: 5 µm), magnifications of corresponding single aggregates are on the right (scale bars: 2 µm).

We visualized the binding of PfnM to ActM filaments microscopically. We first incubated ActM in different molar ratios with FITC-labeled PfnM, stained the mixtures with TRITC-phalloidin after polymerization and viewed the samples under a confocal microscope. ActM polymers appear unaltered in this setup, the binding properties of phalloidin are not visibly influenced by PfnM (Figure 8). We observed a congruence of F-ActM (red) and PfnM (green) derived fluorescence signals indicative of a co-localization. Magnifications of single ActM aggregates show that their entire surface is decorated with PfnM (Figure 8, right). We noticed that the FITC fluorescence of decorated ActM aggregates increases with the amount of FITC-labeled PfnM added, confirming a concentration-dependent binding of PfnM to F-ActM (Figure 8, from top to bottom). Conversely, we found that FITC-labeled PfnM does not stain rabbit actin filaments even if PfnM is present in 4-fold molar excess (Figure S5 B).

**Figure 9:**
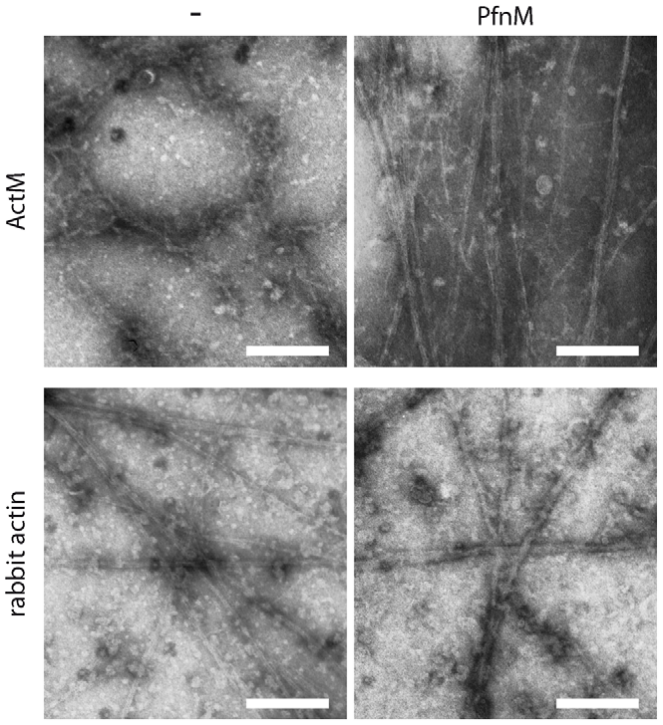
Transmission electron micrographs of ActM and rabbit actin with PfnM. Top row shows ActM filaments without PfnM (left, “-”) and with 4-fold molar excess of PfnM. Bottom row shows rabbit actin controls. Scale bars: 200 nm.

As fluorescence light microscopy does not provide sufficient resolution to display filaments of a width of ∼5 nm, we investigated the effect of PfnM binding on single actin polymers in high resolution by negative-stain electron microscopy. Individual ActM filaments appear as thin, weaving or undulated filaments of lengths between 50 and 300 nm (Figure 9, top left). Agglomerations of irregularly oriented filaments can also be observed, the dimensions of these clusters are typically in the 1–2 µm range. In contrast to that, polymeric ActM is predominately found in straight, cable-like bundles of laterally aligned filaments (Figure 9, top right) in the presence of a 4-fold molar excess of PfnM. These “cables” are much longer than single ActM filaments, frequently measuring more than 2 µm in length. The appearance of rabbit actin filaments, however, remains unaltered by the addition of PfnM (Figure 9, bottom).

To employ a further method to investigate the PfnM-related changes in the ActM polymer structure, we performed SAXS analyses with polymerized ActM after incubation with PfnM. We found that eq. (1) is applicable to describe the SAXS data sufficiently. In accordance with our EM findings, the parallelepiped structure displays an increasing width from 15 nm, to 23 nm, 28 nm and 38 nm for ActM to PfnM ratios of 1∶0, 2∶1, 1∶1 and 1∶2, respectively, whereas the height remains constant at 4.2 nm (Figure S6). No change is observed for the random coil contribution to the scattering as the *R*_g_ remains at 0.7 nm.

Model-free data evaluation methods are useful to check the consistency of the parameters derived from the model curves. Information related to the cross-sectional dimension of fibrils is available from applying the cross-section Guinier law [31]



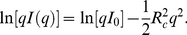
(5)


A straight line in a 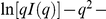
plot yields the radius of gyration of the cross-section *R*_c_. Application of eq. (5) yields 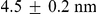
(ActM to PfnM of 1∶0), 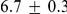 (2∶1), 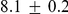 (1∶1) and 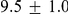 (2∶1) (Figure S6, inset). For comparison we calculated 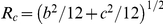 from the model fit parameters as 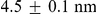 (1∶0), 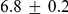 (2∶1), 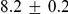 (1∶1) and 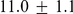 (2∶1). Comparison of the values shows that the *R*_c_-values from Guinier fits and model parameter agree within the experimental range of error.

**Figure 10:**
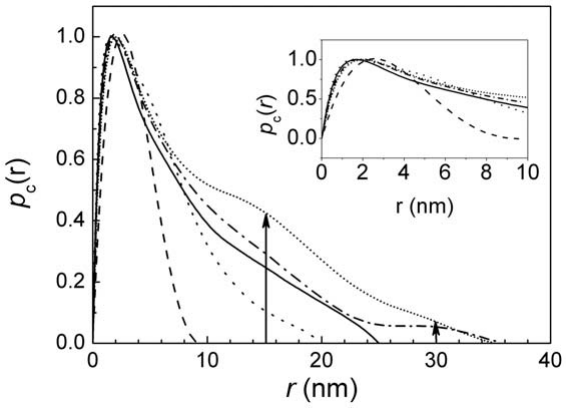
Pair distance distribution function of PfnM-decorated ActM polymers. Cross section pair distribution functions of rabbit F-actin (dashed line), polymerized ActM (dotted) and polymerized ActM with PfnM in molar ratios of 2∶1, 1∶1 and 1∶2 (solid, dash-dotted, shot dotted line, respectively). Maximum dimensions at *p*_c_(*r*)  = 0 are at 9 nm, 20 nm, 25 nm and 35 nm. Inset: Magnification of the region around the maxima of the *p*_c_(*r*).

We obtained further insight into the cross-section filament structure by the pair-distance distribution function (PDDF) of the cross-section, 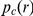, as determined by indirect Fourier transformation of 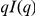 [36]. Multiplying 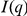 with *q* cancels the contribution from the fibril length to good approximation [37], [38]. The 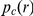 can be interpreted as the electron density weighted number of all possible connections between points within the filament cross-section, thereby providing detailed information on the cross-section (Figure 10). Here, the peak maxima are at ca. 2 nm for all ActM curves while it is around 2.6 nm for rabbit F-actin (Figure 10, inset). These values are approximately half the height of the assumed ActM parallelepiped 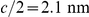 and close to the cylinder radius of rabbit F-actin. The maximum dimensions of the filaments are 9 nm (rabbit), 20 nm (ActM:PfnM 1∶0), 25 nm (2∶1), 35 nm (1∶1; 1∶2) as derived from the distance where 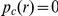. As expected, the 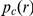 of the cylindrical rabbit F-actin is approximately symmetric, while the 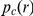 of the ribbon-shaped ActM is highly asymmetric. The contribution of PfnM to the overall shape of the polymer is visible in the appearance of two sub-maxima at around 15 nm and 30 nm (Figure 10, arrows) absent from the PfnM-free sample. Additionally, the maximum filament width in the PfnM-containing samples does not increase in multiples of pure ActM polymers, providing further indication of a PfnM contribution to polymer shape.

The 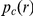 also allows a more precise determination of *R*_c_ as the Guinier plot since it takes information from the total scattering data by



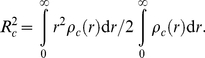
(6)


We determined 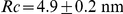
(1∶0), 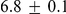 (2∶1), 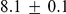 (1∶1) and 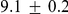 (2∶1) by applying eq. (6) confirming our previous results (Table S1). The model-fit calculations and model-free data evaluation lead to a congruent picture of a ribbon-like structure of polymerized ActM whose average width increases with increasing amount of PfnM during polymerization.

## Discussion

The discovery and characterization of prokaryotic actins has strengthened the current notion of a highly complex bacterial intracellular space. It has become clear that the actins' structural plasticity has resulted in their involvement in a number of essential cellular processes. Cyanobacterial ActM and PfnM, homologs of eukaryotic actin and its actin binding protein profilin, respectively, provide the rare opportunity to analyze the adaptation of one of the most conserved proteins to a foreign cellular environment. Despite having entered the cyanobacterial lineage only rather recently, both ActM and PfnM show a significant degree of change of their amino acid sequence indicative of the evolutionary forces acting on both proteins [7]. Generating GFP-fusion proteins we were able to replicate earlier findings from immunofluorescence microscopy of *Microcystis aeruginosa* in *E.coli* suggesting the presence of a large intracellular shell-like assembly (Figure 1 and S1). ActM forms agglomerations that are not freely diffusible inside *E.coli* cells and frequently localize towards the cell's periphery in a way reminiscent of the ActM distribution in *Microcystis*. Based on the size-dependent diffusion properties of GFP complexes [39] we conclude that ActM-GFP forms smaller oligomeric aggregates when excluded from central or polar regions only (Figure 1 A, B, D). When adopting more distinct shapes such as narrow peripheral layers or traversing bands (Figure 1 C, E) ActM-GFP is most likely aggregated into polymeric structures. As we could verify only one distribution pattern in cells expressing untagged ActM (Figure 1 F), we believe that GFP-ActM does not faithfully represent the protein in all instances. The structural flexibility of the actin molecule may explain why the attachment of fusion-tags possibly restructures the surface of ActM thus limiting further protein-protein interactions including self-aggregation [40]. Additionally, despite the use of a flexible linker peptide, sterical hindrance by the GFP tag itself may be responsible for these limitations. The fusion of the GFP protein to PfnM did not obstruct ActM binding (Figure S4 A), therefore, the strongest indications of the combined capacity of ActM and PfnM to form structures of potential functional relevance stem from experiments with PfnM-GFP (Figure 3). These data suggests that both ActM and PfnM are required for the formation of large enclosures and we propose that sheets or bundles of polymerized ActM together with bound PfnM are their main constituents. The main implications of this proposed model – the ability of ActM to form wide, flat polymers and the binding of PfnM to these – have been tested and verified with different methods in our experiments. We have shown unequivocally that ActM polymerizes into filaments which assemble into extended structures much shorter and wider than typical eukaryotic actin filaments, that ActM and PfnM bind each other specifically and that PfnM has the unusual property of an ActM filament-binding and bundling factor.

Providing low resolution information on the nature of single filaments, SAXS data confirmed our earlier findings from fluorescent phalloidin microscopy and revealed clear differences between ActM and rabbit actin. The scattering curve of ActM shows characteristic features of an elongated polymer which, in contrast to rabbit actin, adopts a ribbon-like shape that can be modeled as a parallelepiped rather than a cylinder. Additionally, the maximum dimension of 20 nm determined by the PDDF analysis may be indicative of an increased tendency of ActM to form higher-order assemblies (Figure 10). The PfnM binding to ActM polymers is also reflected in the SAXS data as the increasing radius of gyration determined with three separate methods proves a concentration-dependent influence of PfnM on the cross section of ActM filaments. The PfnM-dependent increase of the maximum dimensions of ActM polymers seen in the PDDF analysis can be interpreted as augmented bundling of single filaments mediated by PfnM binding. The conspicuous appearance of pair-distance values at *r* = *n* • 15 nm upon the addition of PfnM and their PfnM-dependent rise in probability is an indication of the emergence of a new, wider species of filaments (Figure 10, arrows). Considering the PfnM decoration of filamentous ActM it is very likely that significant amounts of bound PfnM are responsible for this increased width. Supporting this is the low frequency of structures measuring multiples of single ActM polymers whose increase is less pronounced than should be expected for the formation of lateral assemblies solely composed of ActM. This finding is remarkable in that eukaryotic profilin is so far only known as a strict actin monomer-binding protein and has never been found to bind F-actin in larger amounts [35].

The finding that filaments of all ActM:PfnM ratios have identical heights indicates the tendency of single ActM ribbons to align laterally in the presence of PfnM. This is confirmed by the EM images showing the emergence of ActM filament bundles after the addition of PfnM. Conceivably, the static and structural properties of wide co-polymers of filamentous ActM and bound PfnM are more favorable for the formation of a continuous proteinaceous layer than irregular assemblies of individual filaments would be. The heterogeneous shapes visible in cells expressing ActM-GFP alone seem to support that notion. Without the bundling and stabilizing effects of PfnM, the strong curvature observed in the spherical enclosures might be less likely to be established by ActM-GFP filaments alone. Rather, the intracellular localization of single filaments is probably governed by extrusion and displacement effects created by the various components of the *E.coli* cell. The observed concentration of ActM at the inner face of the cell envelope may very plausibly be the result of undirected polymerization along an immobile structure, eventually generating a shell-like pattern.

To accommodate PfnM molecules in ActM filaments we expect either the actin:actin or the actin:profilin contact regions to differ from the eukaryotic archetype. Our data suggest that the former is the case. The ability of PfnM to bind monomeric rabbit actin indicates the high conservation of the actin:profilin interface. In eukaryotes, the profilin binding site becomes inaccessible upon actin polymerization. Accordingly, we have found that PfnM does not bind rabbit F-actin. However, filaments of eukaryotic actin artificially distorted to adopt a ribbon-like shape were shown to provide enough space to allow for profilin binding to the filaments [41], [42]. Combined with the fact that we found ActM filaments to be ribbon-shaped, this appears to be in support of an uncommon ActM:ActM interface. Obtaining high resolution structural information to elucidate the exact PfnM-ActM and ActM-ActM binding geometries should therefore be the objective of future work. Similarly, the relevance of the random-coil structure detected in the ActM filaments remains to be determined.

The natural function of the observed hollow enclosures remains speculative, a contribution to cell stability is an apparent interpretation, additionally, an involvement in bacteriophage related processes can be discussed. The latter is based on the fact that ActM and PfnM are encoded in a genomic island common for bacteriophage mediated genetic transfer and that profilin sequences are found in a number of viral genomes [43], [44].

With this work we have shown that despite being a very slowly evolving protein in eukaryotes, actin displays a remarkable adaptive flexibility once it faces a foreign cellular environment. We provide substantial support to the notion that the high conservation of eukaryotic cytoskeletal proteins is not an inherent feature of their amino acid sequence itself, rather, it is maintained by constrains exerted by the vast multitude of interacting factors [1]. Being placed in an environment devoid of these binding partners and subjected to adaptive pressure, actin and profilin readily developed properties not found in their ancestral eukaryotic proteins. In light of the importance of ABPs in actin evolution and the singular role PfnM – the only known homolog of a eukaryotic ABP in prokaryotes – plays in the formation of distinct ActM aggregates, it is highly probable that in a co-evolutionary process PfnM has directly influenced the reshaping of the ActM sequence. The resultant formation of an intracellular enclosure of potential functional relevance adds to the spectrum of important roles actin proteins play in bacteria and provides further incentive for extensive future work in this field.

## Materials and Methods

### Generation of GFP-fusion proteins

The nucleotide sequence for GFPuv was amplified by PCR from the pGFPuv vector (Clontech) using the primer sequences 5′-TCTAGACTTGAAATGAGTAAAGGAGAA-3′ and 5′-GAATTCTTATTTGTAGAGCTCATCCATGC-3′. The amplicon coded for an N-terminal SRLE-linker peptide and carried an *XbaI* and an *EcoRI* restriction site at the 5′ and the 3′ end, respectively. After subcloning into and *XbaI*/*EcoRI* mediated restriction out of the cloning vector pDRIVE (Qiagen), the *gfpuv*-sequence was ligated into the *XbaI*/*EcoRI*-linearized pBLUESCRIPT II SK (+) vector (Stratagene/Agilent). The *actM* and *pfnM* sequences were amplified from purified genomic DNA from *Microcystis aeruginosa* PCC 7806. Primers used were 5′-GGAGTCTAGAATGAGTGAAATCG-3′ and 5′-CCGCTATAAAACATCTAGAGAAACA-3′ for *actM* and 5′-GTCTAGAATGTATTACGACAGTTACAT-3′ and 5′-TTGTCTAGAAATGCCACGACT-3′ for *pfnM*. The primers were designed to add *XbaI* restriction sites to both ends and to remove the stop-codon. After pDRIVE subcloning and *XbaI* restriction, the thus modified *actM* and *pfnM* sequences were ligated into the GFPuv-carrying, *XbaI*-linearized pBLUESCRIPT II SK (+) vector to yield ActM-SRLE-GFPuv and PfnM-SRLE-GFPuv fusion proteins, respectively.

### Heterologous expression of proteins, cell disruption and antibody generation

Following primers were used for PCR-amplification of the *actM* and *pfnM* genes from purified genomic DNA from *Microcystis aeruginosa* PCC 7806: 5′-CATATGAGTGAAATCGTAATTGATTG-3′ and 5′-GGATCCTTAGAAACATTTTTTATGCAC-3′ for *actM* and 5′-CATATGTATTACGACAGTTACATTG-3′ and 5′-GGATCCTTAAATGCCACGACTTTCTA-3′ for *pfnM*. An *NdeI* and a *BamHI* site (underlined) was added to each gene's 5′ and 3′ end, respectively. After subcloning and excision with *NdeI* and *BamHI*, the *actM* and *pfnM* genes were ligated into the *NdeI-BamHI* linearized pET15b expression vector (Novagen). Expression in *E. coli* BL21 (DE3) cells (Novagen) was induced with 0.5 mM Isopropyl β-D-1-thiogalactopyranoside at OD_600_ of ∼1.0, cells were grown for 4 h at 37°C with shaking at 220 rpm. Cell-free extracts were prepared after pelleting and resuspending expression cultures either in G buffer (5 mM Tris, 0.1 mM CaCl_2_, 0.2 mM ATP, 1 mM NaN_3_, pH 8.0; approx. 1 ml buffer per 100 ml culture volume) supplemented with 40 mM imidazole and 0.5 mM phenyl-methyl-sulphonyl-fluoride (PMSF) for ActM or in native lysis buffer (50 mM NaH_2_PO_4_, 300 mM NaCl, 10 mM imidazole, 0.5 mM PMSF, pH 8.0, Qiagen) for PfnM. After addition of 1 volume of glass beads (d = 0.11 mm and d = 0.18 mm in a 1∶1 ratio, Sartorius), cells were disrupted in a MM2 glass bead mill (Retsch). Cell debris was pelleted by centrifugation. Expressed proteins were purified over a Ni-NTA-agarose matrix following manufacturer's instructions (Qiagen). Wash buffers contained 60 mM and 20 mM imidazole for ActM and PfnM, respectively. The elution buffer contained 250 mM imidazole. Protein purity was determined by SDS-PAGE analysis. To estimate the amount of protein aggregated in inclusion bodies, the pellet remaining after cell disruption was resuspended in an equal volume of denaturating buffer (100 mM Na_2_HPO_4_; 10 mM Tris-HCl; 8 M urea; pH 8.00) and subjected to another round of grinding. Protein levels were assessed via SDS-PAGE and immunoblots. For antibody generation, cell pellets were resuspended in denaturating buffer, 1 mg of denatured protein was used to raise polyclonal antibodies in rabbit serum (Pineda antibody service, Berlin, Germany).

### Co-expression in *E.coli*

GFP-labeled proteins encoded on vectors of the pMB1/ColE1 compatibility group were co-expressed in *E.coli* BL21 (DE3) with proteins encoded on pACYC184 (New England Biolabs) derived vectors with a compatible p15A-type origin of replication. The latter were constructed as follows: Using the primers 5′-TAATACGACTCACTATAGGGG-3′ and 5′-TAGTTCCTCCTTTCAGCAAAA-3′ a region sufficient for heterologous expression of proteins encompassing the complete sequence flanked by and including the T7 promotor and T7 terminator sites were amplified with a proof-reading, blunt-end generating polymerase (“Pfu”; Fermentas) from the pET15b-derived expression vector constructs carrying the desired gene to be co-expressed with the GFP-fusions. This amplicon was then ligated into the pACYC184 vector subjected to *HindIII* restriction and subsequent 5′ overhang fill-in by Klenow-fragment (Fermentas). No inducer of transcription (IPTG) was necessary to produce sufficient amounts of either protein, therefore, to minimize over-expression artifacts, no IPTG was added to the growing cultures.

### Binding and co-elution assays

Proteins expressed heterologously in *E.coli* were attached via 6xHis tag to a Ni-NTA agarose matrix (Qiagen) and washed as if to be purified. Instead of elution, an ensuing equilibration of the loaded agarose matrix with G-buffer (containing 10 mM imidazole for matrix-bound PfnM and 40 mM imidazole for matrix-bound ActM) was followed by the addition of native, non-tagged protein extracts of the potential binding partner in the same buffer. Tag-free PfnM was generated by thrombin mediated cleavage (RECOMT Thrombin CleanCleave Kit, Sigma-Aldrich) of purified 6xHis-tagged PfnM, complete removal of the tag was verified on immunoblots using a monoclonal anti-poly-histidine antibody produced in mouse (clone HIS-1, Sigma-Aldrich). Untagged ActM was expressed in *E.coli* from the pCC2FOS™ fosmid vector (Epicentre/Biozyme) carrying the *actM-pfnM* region from *Microcystis aeruginosa* PCC 7806 in its native genomic environment. The identity of the ∼30 kbp insert generated from purified, fragmented genomic DNA of *Microcystis aeruginosa* PCC 7806 was verified by DNA sequencing (GATC GmbH) and the presence of untagged ActM in native protein extracts was checked on immunoblots. Actin from rabbit skeletal muscle (99%, Cytoskeleton) was purchased. Matrixes were washed three times with G-buffer (supplemented with 20 mM and 60 mM imidazole four matrix-bound PfnM and ActM, respectively) and eluted with 250 mM imidazole in G-buffer.

### Protein blots and immunodetection

Cell-free extracts were separated by SDS-PAGE and blotted onto nitrocellulose membranes (Amersham Pharmacia/GE Healthcare) according to standard procedures [45]. Following commercially available antibodies were used for immunoblots: anti-actin (MA1-744, Affinity BioReagents) and anti-GFP (G1544, Sigma), HRP-coupled anti-rabbit (A9169, Sigma) and anti-mouse (A9044, Sigma). For chemiluminescence signal generation and detection the Pico ECL kit (Pierce/Thermo) and a ChemiDoc XRS+ imaging system (Biorad) were used.

### Fluorescence microscopy

Bacterial cells were fixed in formaldehyde, prepared for immunofluorescence microscopy and either observed with a wide-field deconvolution-based fluorescence microscope as described [7] or with a Zeiss LSM 710 confocal fluorescence microscopy system with an inverted microscope AxioObserver Z.1. The imaging software ZEN 2009 was used for operating the system, image acquisition and processing. For anti-actin immunofluorescence microscopy, a commercially available rabbit polyclonal anti-actin antibody was used (A2066, Sigma) in combination with a TRITC-labeled secondary anti-rabbit antibody (111-025-003, Jackson). *E. coli* cells expressing GFP-tagged proteins were observed alive. After thorough vortexing, cells in liquid culture were diluted 1∶5 in fresh culture medium and a 15 µl sample was directly pipetted onto agarose-coated glass slides. To generate these, 10 µl of a 1% (w/v) solution of low melting point agarose (Biozym) in LB-medium were spread on the slides, air-dried and used within 16 hours.

### FITC-labeling of PfnM

Purified PfnM (1 mg ml^−1^) was conjugated with FITC according to the FluoroTag FITC Conjugation Kit protocol (Sigma). Briefly, 125 µg of FITC was added to 1 mg of protein and allowed to react in 0.1 M carbonat-bicarbonat buffer (pH 9.0) [46] for 1 h at RT. Labeled PfnM was isolated and separated from incorporated FITC with a Sephadex G-25M prepacked column. The molar ratio of incorporated FITC to PfnM (F/P) sufficient to provide satisfying fluorescent intensities was determined to be 1.12.

### Preparation of polymerized actin

Rabbit actin (99% pure; Cytoskeleton) or ActM in G buffer was converted to Mg^2+^-actin by adding 50 µM EGTA and 50 µM MgCl_2_. Mg^2+^-actin was polymerized by adding 1/20 volumes of initiation buffer (2 M KCl, 40 mM Mg Cl_2_).

### Co-polymerization and ultracentrifugation

Purified actin/ActM and PfnM in G-buffer were brought to a concentration of 75 µM and mixed in different molar ratios. Polymerization was initiated and solutions were kept at 4°C over night. Reactions were ultra-centrifuged at 100,000×g, 1 h, 4°C in a Micro-ultracentrifuge MX-150 equipped with an S45-A rotor (Sorvall). Pellet fractions were analyzed by SDS-PAGE and protein-immunoblots. For quantification, bands were identified manually and analyzed using the “Volume” tool of the Image Lab software (BioRad). Manual band detection and subsequent data analyses were performed in triplicates and averaged.

### Phalloidin staining

4 µl of F-actin solution was spread on a poly-L-lysine-coated coverslip. After 2 min, excess fluid was removed, actin filaments were fixed with 2.5% glutaraldehyde in PBS for 5 min, washed 3 times 5 min with PBS and incubated with 50 µl of a 0.165 µM (0.25 U) solution of fluorescence-labeled phalloidin (A488 and TRITC, Molecular Probes/Invitrogen) in PBS for 30 min. Preparations were washed twice in PBS and mounted in 4% (v/v) n-propylgallate dissolved in 87% (v/v) glycerol.

### Electron miscroscopy

Polymerized samples were pelleted by ultracentrifugation, resuspended in F-buffer and spread on poly-L-lysine-coated butvar film and negatively stained with 1% uranyl acetate, supplemented with 2 mM octylglucoside. Samples were imaged in a Philips CM100 operated at 80 kV.

### Small-Angle X-ray Scattering (SAXS)

Actin samples were polymerized and concentrated with concentrator columns (MWCO 7.5 kDa, “Amicon Ultra”, Millipore). The small-angle X-ray scattering measurements of the samples were performed with a SAXSess camera (Anton Paar, Austria). This Kratky type of camera is attached to a laboratory X-ray generator (PW3830, PANalytical), and is operated with a fine focus glass X-ray tube at 40 kV and 50 mA (Cu_Kα_, λ = 0.1542 nm). A focusing multi-layer optic and a block collimator provide a monochromatic primary beam with low background. Samples were filled in a reusable vacuum tight flow cell sample holder. SAXS data (intensity as a function of the scattering vector) was recorded for 1800 s with a CCD detection system in a *q*-range of 0.08 to 6.0 nm^−1^ (Anton Paar). The scattering vector is defined in terms of the scattering angle, *θ* and the wavelength, *λ* of the radiation, thus *q* = 4*π*/*λ*sin(*θ*). For clarity, the angle between incident and scattered beam is 2*θ*. The two-dimensional intensity data was converted to one-dimensional data with CCDQuant software (Anton Paar). The temperature of 25°C was controlled with a TCS 120 sample holder (Anton Paar) with an accuracy of ±0.2°C. A reusable capillary was used for all measurements to attain the same scattering volume and background contribution. The resulting scattering curves were corrected for the contribution of the suspension medium (water) and the glass capillary. Furthermore, the data was de-smeared using the length profile of the primary beam [47] with SAXSQuant (Anton Paar).

## Supporting Information

Figure S1 **Localization of ActM in environmental samples of** ***Microcystis*** **.** Immunofluorescence microscopy with an anti-actin antibody and FITC label (green channel) suggests a shell-like distribution of ActM in the cell. Note the absence of specific FITC signal in the cells of the bottom part indicative of the heterogeneity of environmental samples. Autofluorescence of the thylakoid system is shown in the red channel. Scale bars: 5 µm.
(TIF)

Figure S2 **Solubility assay.** Immunodetection with cell extracts of *E.coli* co-expressing PfnM-GFP and ActM. Actin (left) and PfnM-GFP (middle and right) are found predominately in the soluble fraction (SF) as opposed to aggregating in inclusion bodies (IB).
(TIF)

Figure S3 **Binding and co-elution of rabbit actin and immobilized PfnM.** Protein blots and immunodetection of final wash (W) and eluate (E) are shown; employed antibody is indicated at the bottom. Molecular weights are 43 kDa for actin and 17 kDa for PfnM.
(TIF)

Figure S4 **GFP-fusion proteins co-elution assay.** Immunodetection of identical samples from binding assays with mobile, soluble PfnM-GFP and immobilized ActM (A) and mobile, soluble ActM-GFP on immobilized PfnM (B). Soluble ActM-GFP does not co-elute (“E”) with immobilized PfnM (17 kDa and 35 kDa). Instead, a 65 kDa band representing ActM-GFP is found exclusively in the flow-through fraction (“FT”). “W1”, “W3”: First and last washing step, respectively. PfnM-GFP (42 kDa) co-elutes with ActM (40 kDa; “E”); the final wash fraction is also shown (“W”). Antibodies used for detection are indicated at the bottom of the figure.
(TIF)

Figure S5 **Assessing the binding of PfnM to rabbit actin filaments.** Polymerization and ultracentrifugation of rabbit actin and PfnM (A). SDS-PAGE of pellet fractions of different actin:PfnM ratios (indicated at the top). The relative amount of PfnM in the pellet is indicated at the bottom. FITC-labeled PfnM (“PfnM-FITC”, green) was added to phalloidin-TRITC stained rabbit F-actin (“actin-TRITC”, red, B) in a 4-fold molar excess. Scale bars: 2 µm.
(TIF)

Figure S6 **SAXS pattern of ActM polymerized in the presence of PfnM.** Molar ratios of ActM to PfnM were 2∶1, 1∶1 and 1∶2 (symbols). The best fits according to eq. (1) are shown (solid lines). The widths of the parallelepiped are *b* = 23 nm, 28 nm and 38 nm for ActM to PfnM ratios of 2∶1, 1∶1 and 1∶2, respectively. The height is the same for all ratios at *c* = 4.2 nm and the length is held constant at *a* = 500 nm. The radius of gyration for the random-coil contribution is 0.7 nm. Inset: Cross-section Guinier plots of the same data (symbols) and Guinier fits (solid lines) reveal *R*_c_-values of 6.7, 8.1 and 9.5 for 2∶1, 1∶1 and 1∶2 ActM to PfnM radios.
(TIF)

Table S1 **Parameters of polymerized ActM at different ActM to PfnM ratios.** Curve fit values for the parallelepiped values are the width *b*. The length *a* was 500 nm and the height *c* was 4.2 nm for all ActM to PfnM ratios. Different methods were used for the determination of the cross section radius of gyration, *R_g_.*
(PDF)
