# Supplementary figures and images for: Unique Properties of Eukaryote-Type Actin and Profilin Horizontally Transferred to Cyanobacteria
Source: PLoS One. 2012 Jan 10;7(1):e29926. doi: 10.1371/journal.pone.0029926 (PMC3254629; doi:10.1371/journal.pone.0029926)

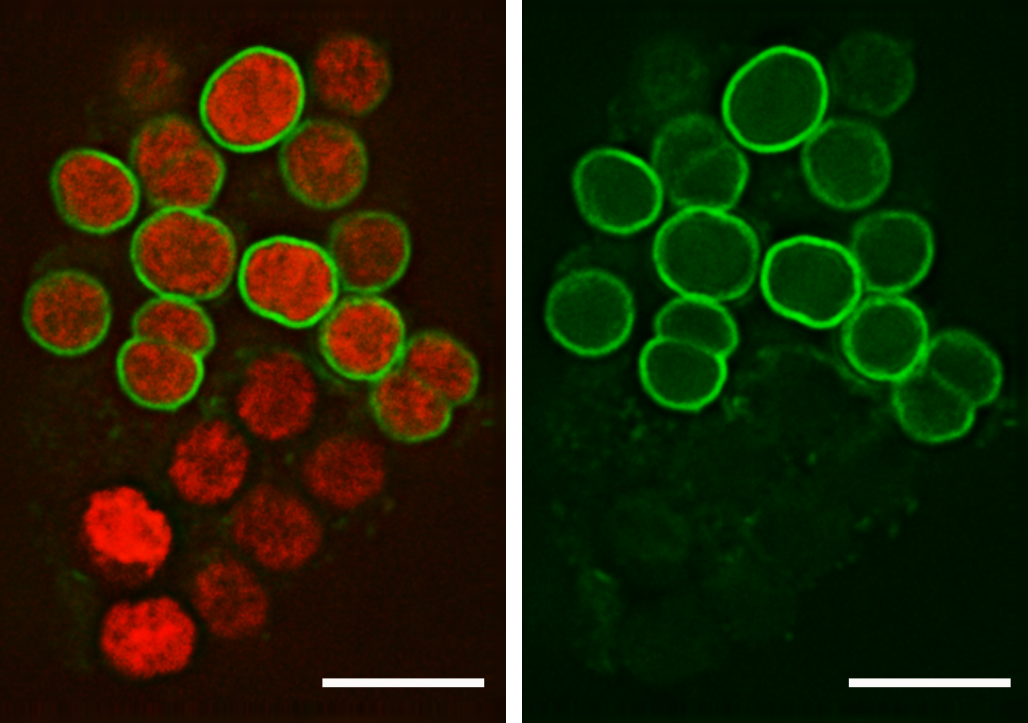

Supplement: Figure S1 — Localization of ActM in environmental samples of Microcystis . Immunofluorescence microscopy with an anti-actin antibody and FITC label (green channel) suggests a shell-like distribution of ActM in the cell. Note the absence of specific FITC signal in the cells of the bottom part indicative of the heterogeneity of environmental samples. Autofluorescence of the thylakoid system is shown in the red channel. Scale bars: 5 µm. (TIF) [file pone.0029926.s001.tif]

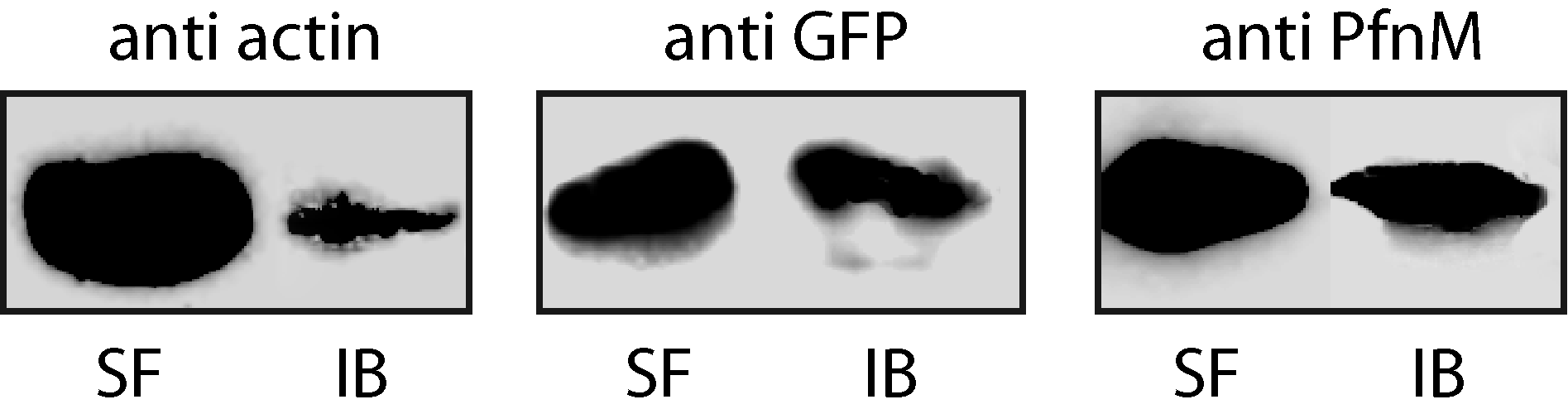

Supplement: Figure S2 — Solubility assay. Immunodetection with cell extracts of E.coli co-expressing PfnM-GFP and ActM. Actin (left) and PfnM-GFP (middle and right) are found predominately in the soluble fraction (SF) as opposed to aggregating in inclusion bodies (IB). (TIF) [file pone.0029926.s002.tif]

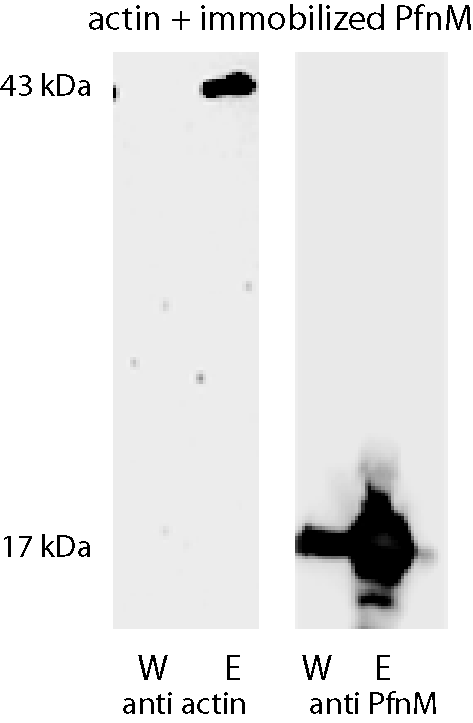

Supplement: Figure S3 — Binding and co-elution of rabbit actin and immobilized PfnM. Protein blots and immunodetection of final wash (W) and eluate (E) are shown; employed antibody is indicated at the bottom. Molecular weights are 43 kDa for actin and 17 kDa for PfnM. (TIF) [file pone.0029926.s003.tif]

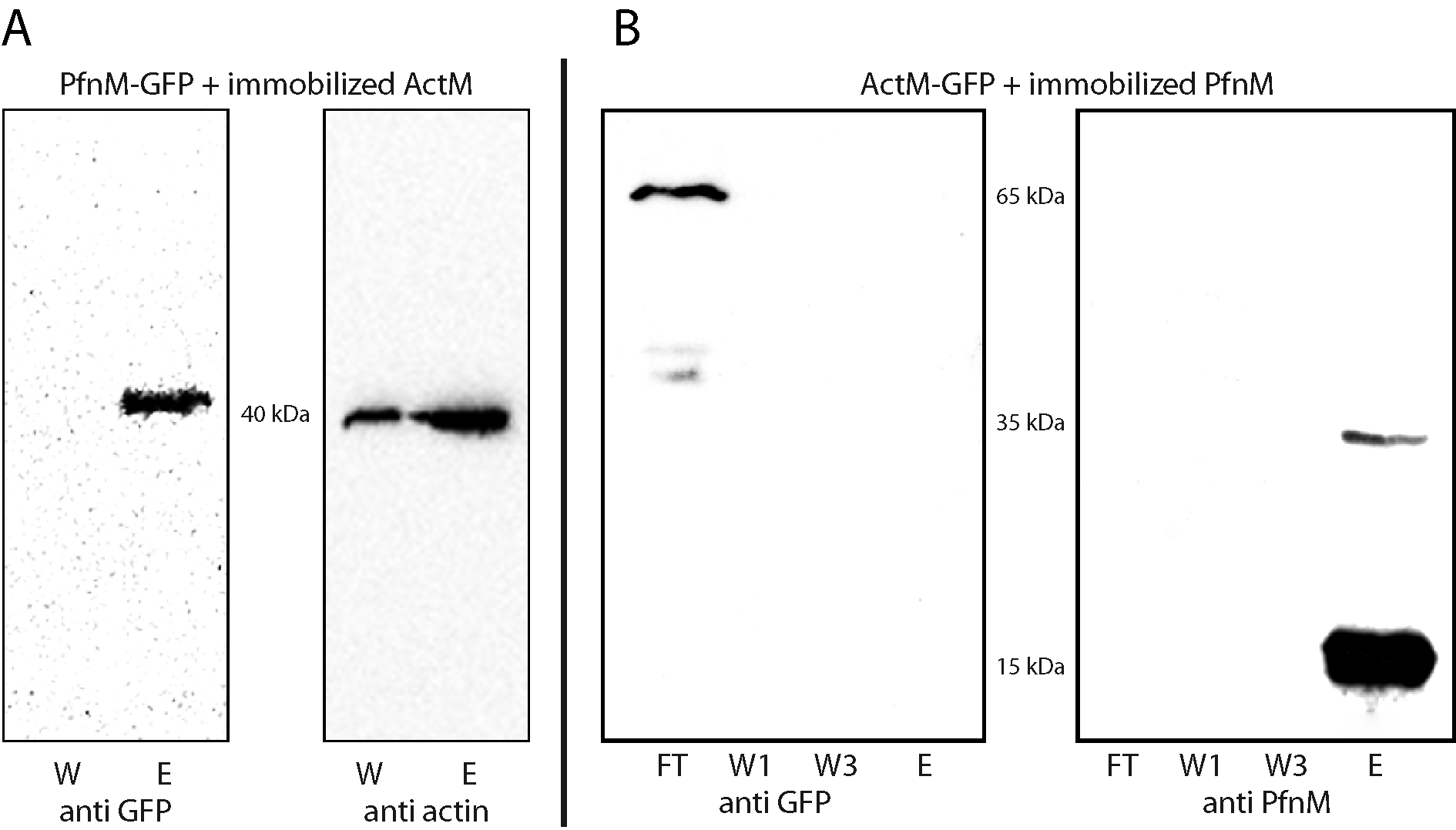

Supplement: Figure S4 — GFP-fusion proteins co-elution assay. Immunodetection of identical samples from binding assays with mobile, soluble PfnM-GFP and immobilized ActM (A) and mobile, soluble ActM-GFP on immobilized PfnM (B). Soluble ActM-GFP does not co-elute (“E”) with immobilized PfnM (17 kDa and 35 kDa). Instead, a 65 kDa band representing ActM-GFP is found exclusively in the flow-through fraction (“FT”). “W1”, “W3”: First and last washing step, respectively. PfnM-GFP (42 kDa) co-elutes with ActM (40 kDa; “E”); the final wash fraction is also shown (“W”). Antibodies used for detection are indicated at the bottom of the figure. (TIF) [file pone.0029926.s004.tif]

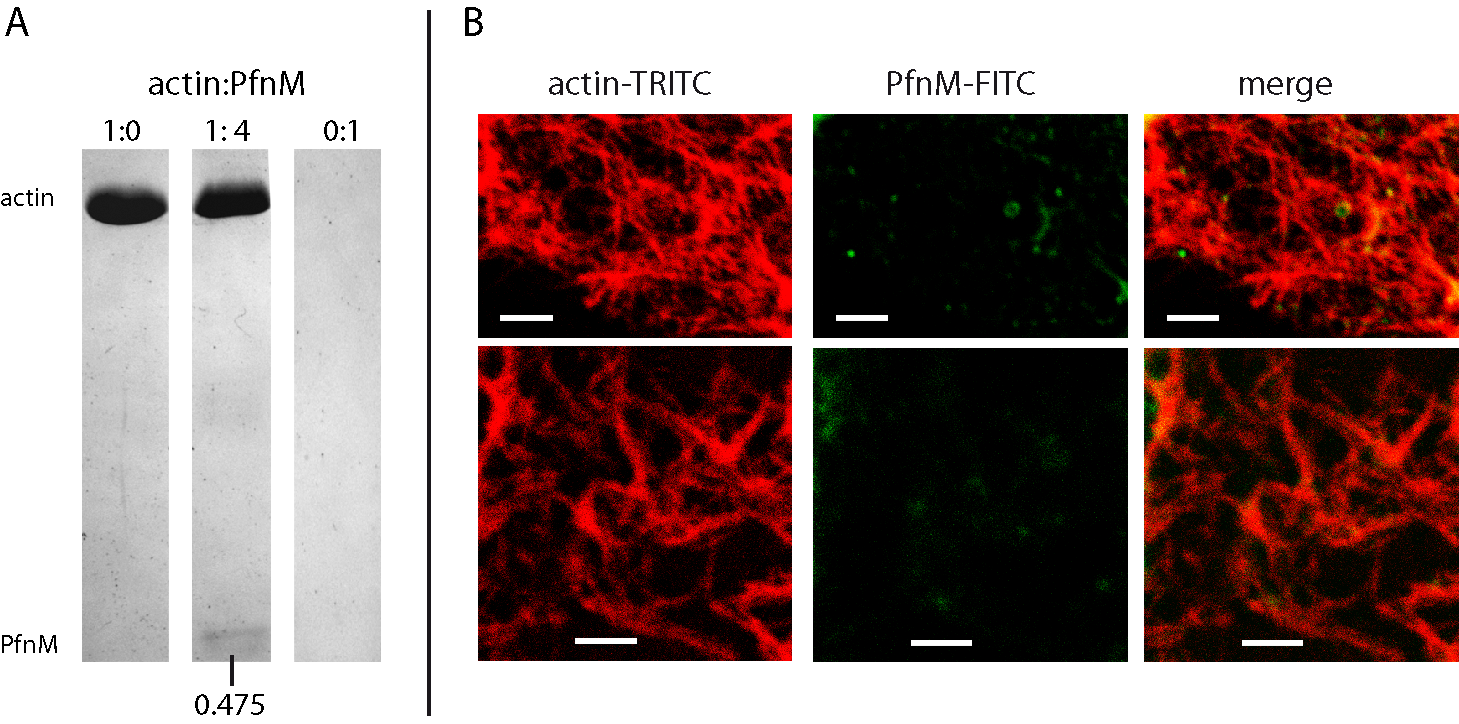

Supplement: Figure S5 — Assessing the binding of PfnM to rabbit actin filaments. Polymerization and ultracentrifugation of rabbit actin and PfnM (A). SDS-PAGE of pellet fractions of different actin:PfnM ratios (indicated at the top). The relative amount of PfnM in the pellet is indicated at the bottom. FITC-labeled PfnM (“PfnM-FITC”, green) was added to phalloidin-TRITC stained rabbit F-actin (“actin-TRITC”, red, B) in a 4-fold molar excess. Scale bars: 2 µm. (TIF) [file pone.0029926.s005.tif]

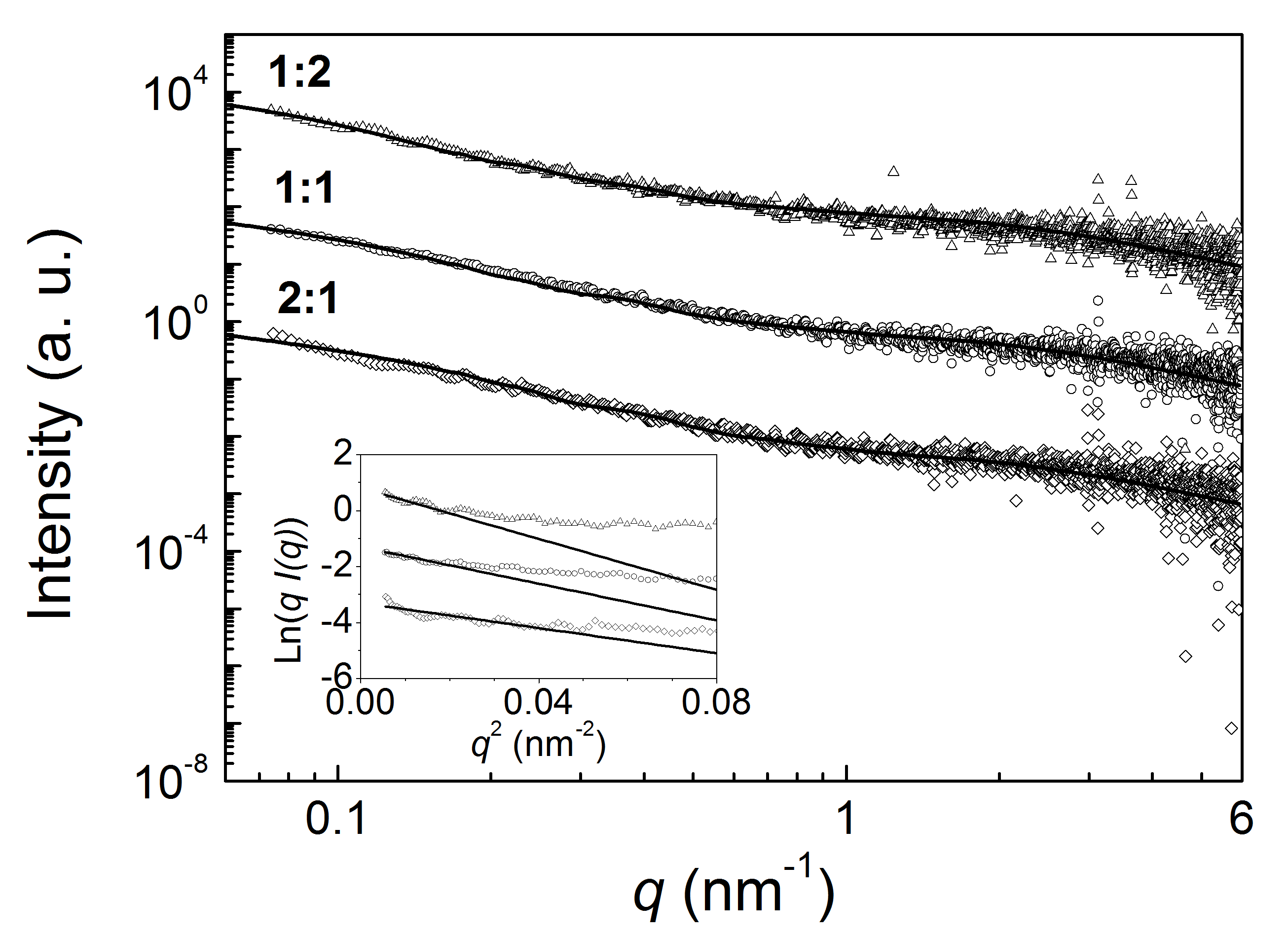

Supplement: Figure S6 — SAXS pattern of ActM polymerized in the presence of PfnM. Molar ratios of ActM to PfnM were 2∶1, 1∶1 and 1∶2 (symbols). The best fits according to eq. (1) are shown (solid lines). The widths of the parallelepiped are b = 23 nm, 28 nm and 38 nm for ActM to PfnM ratios of 2∶1, 1∶1 and 1∶2, respectively. The height is the same for all ratios at c = 4.2 nm and the length is held constant at a = 500 nm. The radius of gyration for the random-coil contribution is 0.7 nm. Inset: Cross-section Guinier plots of the same data (symbols) and Guinier fits (solid lines) reveal R c-values of 6.7, 8.1 and 9.5 for 2∶1, 1∶1 and 1∶2 ActM to PfnM radios. (TIF) [file pone.0029926.s006.tif]
