# Supplementary material for: Unique Properties of Eukaryote-Type Actin and Profilin Horizontally Transferred to Cyanobacteria
Source: PLoS One. 2012 Jan 10;7(1):e29926. doi: 10.1371/journal.pone.0029926 (PMC3254629; doi:10.1371/journal.pone.0029926)
Supplement: Table S1 — Parameters of polymerized ActM at different ActM to PfnM ratios. Curve fit values for the parallelepiped values are the width b. The length a was 500 nm and the height c was 4.2 nm for all ActM to PfnM ratios. Different methods were used for the determination of the cross section radius of gyration, Rg. (PDF) [file pone.0029926.s007.pdf]

Table S1. Parameters of polymerized ActM at different ActM to PfnM ratios.

Curve fit values for the parallelepiped values are the width *b*. The length *a* was 500 nm and the height *c* was 4.2 nm for all ActM to PfnM ratios. Different methods were used for the determination of the cross section radius of gyration, R_g.

Ratio<br>ActM : PfnM	*b* [nm]<br>Model	$R_{g,c}$[a] [nm]<br>Model	$R_{g,c}$[b] [nm]<br>Guinier	$R_{g,c}$[c] [nm]<br>PDDF
1:0	15 ± 2	4.5 ± 0.1	4.5 ± 0.2	4.9 ± 0.2
1:2	23 ± 1	6.8 ± 0.2	6.7 ± 0.3	6.8 ± 0.1
1:1	28 ± 2	8.2 ± 0.2	8.1 ± 0.2	8.1 ± 0.1
2:1	38 ± 4	11.0 ± 1.1	9.5 ± 1.0	9.1 ± 0.2

[a]Values were calculated from the curve fit parameters *b* and *c*. [b]Values were calculated from cross-section Guinier law. [c]Values were calculated from the cross section pair distance distribution function $p_c(r)$.
